# The ideal treatment timing for diabetic retinopathy: the molecular pathological mechanisms underlying early-stage diabetic retinopathy are a matter of concern

**DOI:** 10.3389/fendo.2023.1270145

**Published:** 2023-11-09

**Authors:** Wen-Jie Sun, Xue-Dong An, Yue-Hong Zhang, Xue-Fei Zhao, Yu-Ting Sun, Cun-Qing Yang, Xiao-Min Kang, Lin-Lin Jiang, Hang-Yu Ji, Feng-Mei Lian

**Affiliations:** ^1^Guang’anmen Hospital, China Academy of Chinese Medical Sciences, Beijing, China; ^2^China Academy of Chinese Medical Sciences, Beijing, China; ^3^Beijing University of Chinese Medicine, Beijing, China

**Keywords:** diabetic retinopathy, non-proliferative phase, neurovascular unit, blood-retinal barrier, inflammation, pathological changes, treatment

## Abstract

Diabetic retinopathy (DR) is a prevalent complication of diabetes, significantly impacting patients’ quality of life due to vision loss. No pharmacological therapies are currently approved for DR, excepted the drugs to treat diabetic macular edema such as the anti-VEGF agents or steroids administered by intraocular route. Advancements in research have highlighted the crucial role of early intervention in DR for halting or delaying disease progression. This holds immense significance in enhancing patients’ quality of life and alleviating the societal burden associated with medical care costs. The non-proliferative stage represents the early phase of DR. In comparison to the proliferative stage, pathological changes primarily manifest as microangiomas and hemorrhages, while at the cellular level, there is a loss of pericytes, neuronal cell death, and disruption of components and functionality within the retinal neuronal vascular unit encompassing pericytes and neurons. Both neurodegenerative and microvascular abnormalities manifest in the early stages of DR. Therefore, our focus lies on the non-proliferative stage of DR and we have initially summarized the mechanisms involved in its development, including pathways such as polyols, that revolve around the pathological changes occurring during this early stage. We also integrate cutting-edge mechanisms, including leukocyte adhesion, neutrophil extracellular traps, multiple RNA regulation, microorganisms, cell death (ferroptosis and pyroptosis), and other related mechanisms. The current status of drug therapy for early-stage DR is also discussed to provide insights for the development of pharmaceutical interventions targeting the early treatment of DR.

## 1 Introduction

The International Diabetes Federation 2021 Congress noted that 537 million adults (aged 20-79 years) worldwide suffer from diabetes mellitus (DM), which is expected to reach 784 million by 2045. A common complication of DM is diabetic retinopathy (DR), which is one of the leading causes of blindness over the age of 50 (1). DR can be classified into two key stages based on severity: the early non-proliferative stage and the late proliferative stage. The pathological changes, which are predominately neovascularization in the proliferative phase, are the first distinguishing factor between proliferative DR (PDR) and non-proliferative DR (NPDR) (2). At the cellular level, it is the endothelial cells that predominate in proliferation. In contrast, in the non-proliferative phase, it is characterized by microangiomas and hemorrhages (3, 4). The primary pathological alterations at the cellular level include loss of pericytes, neuronal cell death, loss of components, and functional destruction of the retinal neuronal vascular unit, which contains pericytes and neuronal components (5, 6). It is challenging to distinguish between the two in terms of the mechanism of action, it is currently difficult to distinguish between the two, because mechanisms such as inflammation (7) and oxidative stress (OS) (8) are involved in the onset and development of both proliferative and non-proliferative phases (**Table 1**).

**Table 1:** The difference between different stages of DR.

Stage	Pathological changes	Cellular level	Mechanism
NPDR	Microhemangioma and hemorrhage	The proliferation of endothelial cells	Inflammation and oxidative stress
PDR	Neovascularization	Pericyte loss, neuronal cell death, component depletion and functional impairment of the retinal neurovascular unit, including pericytes and neurons	

No pharmacological therapies are currently approved for DR, excepted the drugs to treat diabetic macular edema (DME) such as the anti-vascular endothelial growth factor (VEGF) or steroids administered by intraocular route. Anti-VEGF agents may reduce the risk of vision loss to some extent, but they do not eliminate it. In severe cases of DR, ophthalmologists with training in laser surgery and laser vision correction are needed (9, 10). Only a small percentage of patients will be able to improve their vision (8, 11), while severe cases will require significant medical resources, including surgery and lasers. Nevertheless, the global burden of DR is expected to remain high until 2045 and PDR remains the leading cause of moderate and severe vision loss in most countries (12). In consequence, patients with DR progressing to the proliferative stage are at high risk of losing their vision. By stopping or delaying the progression of DR at a very early stage, the quality of life of patients can be improved and healthcare costs can be reduced.

The purpose of this review is to discuss the mechanisms of action around early pathological changes associated with NPDR to provide a reference for the development of early therapeutic drugs for DR.

## 2 Pathological changes in the early stages of DR

**Figure 1:**
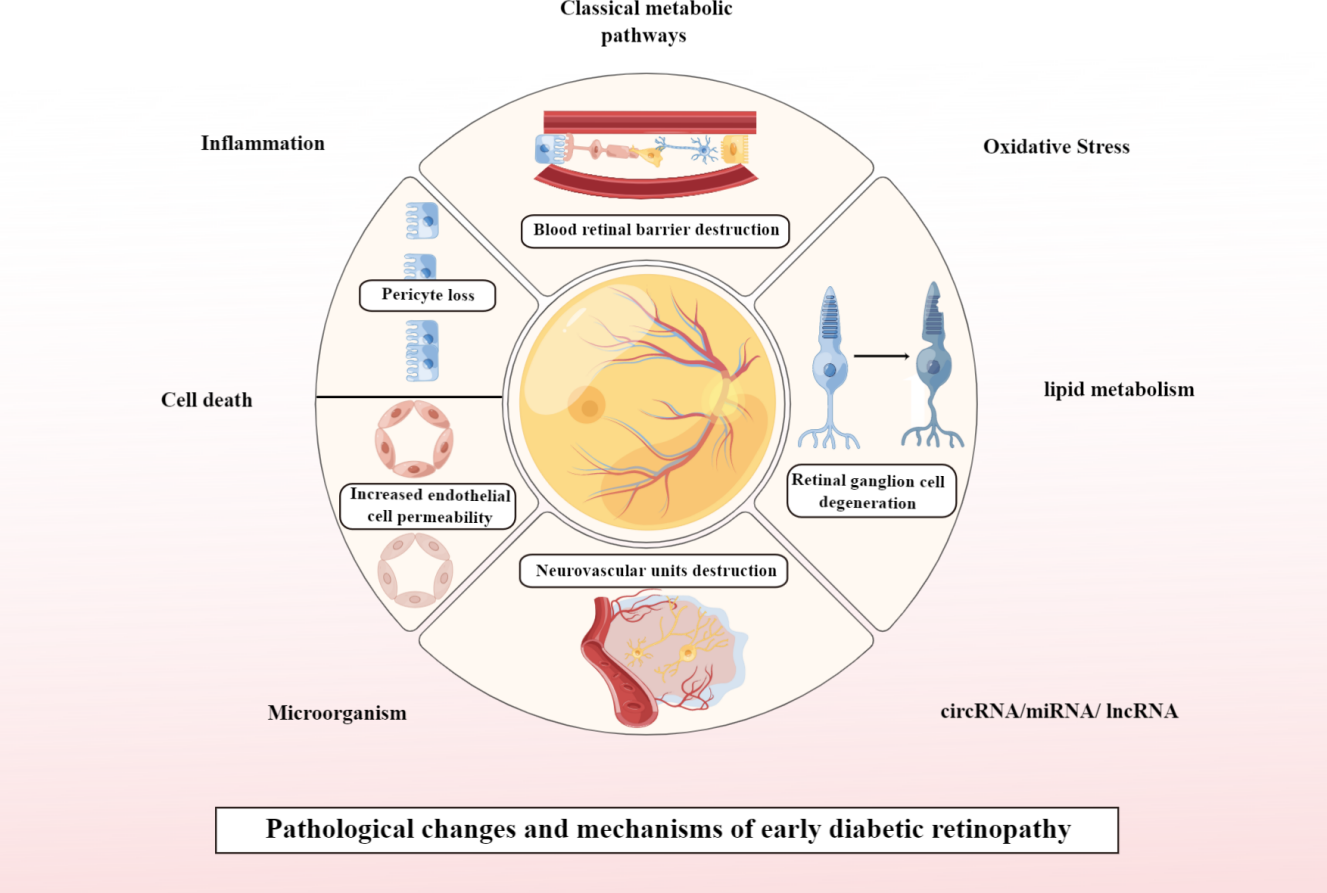
Pathological changes and mechanisms of early DR. Created By Figdraw. circRNA, Circular RNA; miRNA, microRNAs; IncRNA, Long noncoding RNA.

Currently, DR is considered to be a progressive neurovascular disease, with early DR pathology presenting with neurological and vascular abnormalities (13). If it progresses to PDR, the pathological changes are predominantly vascular abnormalities. Pathological changes that play a key role in the early stages of DR include loss of pericytes associated with vascular changes and increased endothelial cell permeability, leading to disruption of the blood-retinal barrier (BRB). In addition, DM also impairs the function of retinal neuronal cells before the BRB is significantly altered, and the pathological changes associated with neurodegeneration in early DR mainly involve ganglion cell degeneration. In addition, destruction of neurovascular units is a concept that is currently valued in early studies of DR and is therefore addressed separately in this article. This is shown in **Figure 1**.

The loss or failure of pericytes in the diabetic retina is a central mechanism for loss of vascular integrity (14, 15) and is one of the hallmarks of DR. As a result, it is hypothesized to initiate or trigger several pathological features (16), such as microaneurysm formation, abnormal leakage, edema, and ischemia, which are thought to trigger proliferative neovascularization of the retina (17, 18). Also typical of the early stages of the disease are disruption of the vascular endothelium and the breakdown of the BRB (19, 20). Physiologically, the endothelium is surrounded by pericytes, which share the same basement membrane and have a variety of connections, including tight junctions and needle-slot complexes, ensuring the structural integrity of the vessel wall. The platelet-derived growth factor receptor-β on the surface of pericytes binds to platelet-derived growth factor-B, which is secreted by the new endothelium. Pericytes can also secrete angiotensin-1 and transforming growth factor-β (TGF-β), which regulate endothelial cell function via the paracrine pathway (21). The clinical study has validated TGF-β as a biomarker for the progression of DR upon treatment with anti-VEGF (22). Microvascular physiology is maintained by the structural and functional support provided by pericytes and endothelial cells. Pericytes are involved in the formation of the BRB as a filter for retinal cells, protecting them against harmful factors in the blood (23). When pericytes constract the cytosol, blood flow is regulated and the diameter of vessels is altered (24). Adipocytes, chondrocytes, and phagocytes, which play a role in tissue repair, can differentiate into pericytes, which are mesenchymal stem cells (25).

With the fresh knowledge of retinal pathology, retinal neurodegenerative lesions precede clinically detectable microvascular damage (26) occurring in the early stages of DR (27, 28). One of the initial steps in the etiology of DR may be the synaptic neurodegeneration of retinal ganglion cells (RGCs). The only projection neurons in the neural retina that take in, process, and send visual information to the brain from upstream retinal neurons in the visual circuit are RGCs. About 40 different RGC types carry out the function of RGCs and project to various destinations in the central brain (29). However, they are highly vulnerable to external damage, mainly because of their restricted trajectory and space. RGCs have limited capacity for endogenous regeneration after injury, hence apoptosis can lead to permanent vision loss. RGCs are the most delicate neurons in the retina and are particularly sensitive to stress brought on by DM in the early stages of nerve development (30). Abnormal glycogen synthase kinase-3 activation under DM-induced metabolic stress causes tau hyperphosphorylation and -linked protein downregulation, which causes mitochondrial damage and synaptic neurodegeneration prior to RGC apoptosis (31). The neurovascular unit also represents an early pathological change, comprising neurons, glial cells and vascular cells. Neurovascular function in the retina is closely linked to BRB’s structural integrity. This is done to support retinal metabolism by maintaining a stable intraretinal environment and regulating local blood flow. The retinal neurovascular unit can be damaged by DM-induced retinal dysfunctions (28). Neurons in the retina include photoreceptors, horizontal cells, bipolar cells, anaplastic cells, and ganglion cells. Research has shown that intraretinal neurons are essential to the deep retinal vascular plexus as they direct superficial blood vessels to deeper layers. Neurons and glia in the retina, for example, produce VEGF to promote retinal vascular development (32). Astrocytes and Müller cells (MGCs) ominate the glial cells of the retina, and microglia and oligodendrocytes provide support and barrier roles for the the retina (33). Glial cells surround neurons and form the main defense and homeostatic structures of retinal neurons. It is the glial cells that deal with the communication between neurons and vascular systems, and these cells are crucial in regulating the function between the two (34). With cell end feet tightly attached to the basement membrane of capillary endothelial cells and pericytes, astrocytes serve a key role in establishing the superficial retinal vascular system. Local neuronal cells can build connections with vascular cells as a result, thus supporting peripheral neurons’ functional and metabolic functions. They are responsible for neuronal metabolism when they mediate between the vasculature and neurons. Additionally, MGCs act as glial transmitters, release neurotrophic factors, regulate extracellular space, maintain the internal BRB, maintain neurotransmitter circulation, protect the retinal structure and direct light to photoreceptors, and maintain the structure of the retina (35). Microglia, distributed in the inner retinal layer at rest, maintain the homeostasis of the intraretinal environment. Activated microglia are divided into pro-inflammatory and anti-inflammatory types upon pathological stimulation. Pro-inflammatory microglia migrate and proliferate more at the site of damage and secrete pro-inflammatory factors, whereas anti-inflammatory microglia are mainly phagocytic, with little variation in their ability to migrate and proliferate.

Both neurodegeneration and microvascular abnormalities occur in the early stages of DR. Therefore, we take the pathological mechanisms surrounding these two as the early pathological landing point of DR, and also add the frontier directions for future development based on the summary of the mechanisms of early DR pathological changes to provide the reference of ideas for the subsequent early treatment of DR.

## 3 Mechanisms of early pathological changes in DR

These mechanisms mentioned in chapter 3.1 are well established and are currently recognised. For the mechanisms mentioned in chapter 3.2 and later, they have been accepted by quite a few scholars, but still need to be further explored and deepened, and there is still much room for exploration.

### 3.1 Classical metabolic pathways in the pathological changes of DR

This disease is primarily triggered by multiple metabolic pathways, including the polyol pathway, the AGEs pathway (36), the hexosamine biosynthesis pathway (HBP), the phosphatidylinositol signaling pathway (PKC).

Either diabetic rat retinal neurons or high glucose (HG) cultured human retinal endothelial cells show increased aldose reductase immunoreactivity in the polyol pathway (37). The formation of three phosphoglucose results from the activation of the polyol pathway and the phosphorylation of fructose converted with sorbitol. This glycosylation results in the formation of three deoxyglucose molecules, which are released as AGEs (38). These products develop irreversibly when dicarbonyl compounds of strong glycosylation are used in their manufacture, such as glyoxal and methylglyoxal (39). There is now clinical evidence of elevated levels of AGEs in patients with DR (40). In the pathophysiology of DR, AGE interacts with the RAGE receptor to activate NADPH oxidase, which results in reactive oxygen species (ROS). It also activates noncanonical nuclear factor-kappaB (NF-κB), which leads to apoptosis and increased expression of inflammatory cytokines and adhesion molecules in peri-retinal cells (41). The production of large amounts of ROS by the host in response to HG can inhibit the activity of 3-phosphoglyceraldehyde dehydrogenase, resulting in glycolytic products that affect the HBP pathway (42). In addition to inducing OS, hydrogen peroxide production from activated hexosamine also increases glucosamine production (42). PKC-α, PKC-β, PKC-δ, and PKC-ϵ of the PKC family are involved in DR pathogenesis and are activated in this process (43). Hyperglycaemia promotes diacylglycerol synthesis to activate the PKC pathway (44). In vascular cells such as endothelial, pericytes, and thylakoids, PKC increases NADPH oxidase activity and promotes ROS production, causing retinal capillary damage and dysfunction of vascular endothelial cells (45). Alterations in renin-angiotensin system-mediated vascular permeability may cause BRB disruption in DR. Ang II activates PKC and enhances NADPH oxidase activity, increasing ROS production and leading to direct retinal damage (46, 47).

### 3.2 Inflammation and its processes, including neutrophil extracellular traps and inflammatory mediators, permeate the development of disease

**Figure 2:**
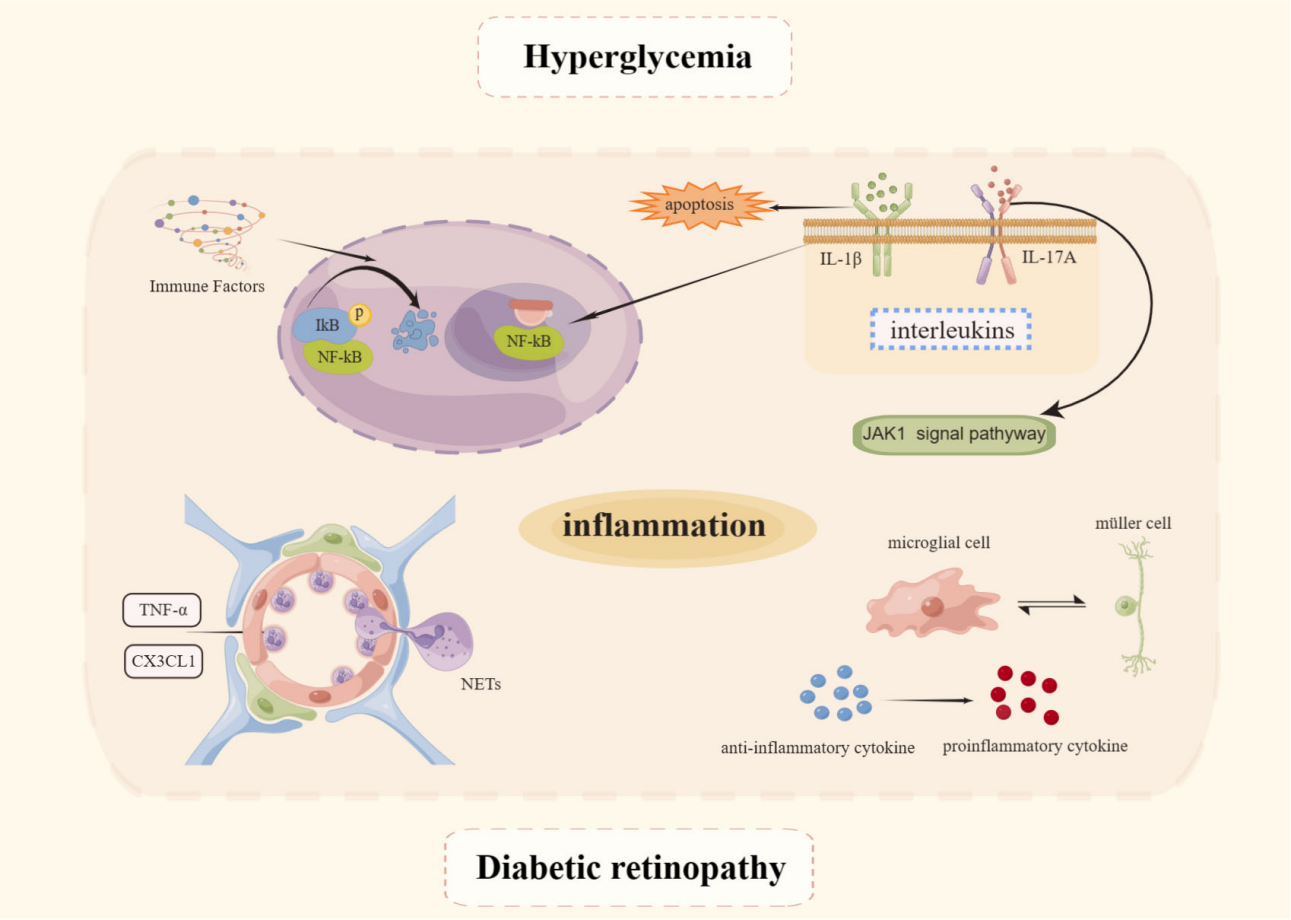
Mechanisms of inflammation in DR. Created By Figdraw. IL, Interleukin; TNF-α, Tumor necrosis factor-α; NET, Neutrophil extracellular trap.

Inflammation is present not only throughout DM but also throughout the entire course of DR (48) which mechanism is depicted in **Figure 2**. Evidence is now available that levels of several inflammatory factors such as interleukin (IL)-1β, IL-6, IL-8, and tumor necrosis factor-α (TNF-α) become elevated during the NPDR phase (49). The NF-κB signaling pathway plays a pivotal role in immunity, inflammation, cell proliferation, and apoptosis leading to inflammation. The protein induces apoptosis in retinal cells, disrupts the BRB, from which it exerts an influence in the development of DR (50). NF-kB core transcription factor, consisting of p65 and p50 heterodimers, is inactive in cells by binding to inhibitors of the natural inhibitor family kinase B-α (IkBα). When stimulated by immune factors, IκB is readily phosphorylated, then ubiquitinated, and finally IκB degradation dissociates from NF-κB and activates it. NF-κB becomes free, switches from the cytoplasm to the nucleus, binds to the corresponding site on the target gene, and intervenes in the inflammatory response (51). The HG environment can induce phosphorylation of NF-κB p65 sites in human retinal endothelial cells and MIO-M1 MGCs, and promotes the release of inflammatory mediators such as intercellular adhesion molecule-1 (ICAM-1), vascular cell adhesion molecule-1 (VCAM-1), and COX-2 only when the pro-inflammatory factors TNF-α and IL-1β are added. This also suggests that the NF-κB signaling pathway plays a role in the inflammatory process, while occupying an important position in the early stages of sarcoplasmic reticulum pathogenesis (52).

Because of the many interleukins involved in the pathogenesis and progression of DR, it is possible to use low serum levels of IL-1Ra as an indicator of DR progression (53). IL-1β, as a pro-inflammatory cytokine, can cause direct cellular damage, resulting in apoptosis of retinal pigment epithelial cells and impairment of photoreceptor function (54). Next, IL-1β can activate NF-κB and OS, leading to apoptosis of peripapillary retinal cells and increased endothelial cell permeability (55, 56). A variety of inflammatory factors and chemokines are expressed by target cells in response to IL-17A’s pro-inflammatory function. By disrupting distribution of ZO-1 in cells, reducing trans-epithelial electrical resistance, and increasing permeability to FITC tracer *in vitro*, interleukin-17A causes BRB impairment via the JAK1 signaling pathway (57). As well as degrading VE-calmodulin through the activation of MyD88, NF-B, and PAR2, it can also enhance the production of neutrophil elastase (NE) (58).

Others, such as TNF-α, a pro-inflammatory cytokine, increase leukocyte adhesion and endothelial injury (59), causing leakage from restricted vessels by increasing endothelial cell permeability and decreasing the distribution of tight junction proteins (60). The chemokine CX3CL1 (part of the CX3C family) captures circulating leukocytes directly and binds to its receptor CX3CR1 to promote migration, adhesion, and proliferation of inflammatory cells (61), and induces calcium ions to accumulate and migrate to the inflamed vessel wall (62). There is a positive correlation between ICAM-1 and VCAM-1 levels and retinal damage (63, 64), while DM increased the expression of ICAM-1 (65).

Leukocytes are known to perform a key role in the host inflammation process. Hyperglycemia activates circulating leucocytes in the early stages of DR, which promotes leucocyte adhesion to the vessel wall, contributing to the inflammatory response (66, 67) and driving the development of DR (68, 69). It has been shown that leukocyte aggregation and adhesion alter hemodynamic function, in contrast to the neutrophil extracellular traps (NETosis) which occurs when large groups of neutrophils adhere to endothelial cells in a reticular fashion (70). Although NETosis can remodel unhealthy blood vessels at the incipient phase, excessive reticular scaffold formation can adversely affect circulation, leading to nonperfusion formation and to tissue ischemia and hypoxia (71). Currently, clinical studies have also shown elevated release products of NETs in patients with type 2 diabetes (T2DM) (72), as well as in patients with DR (73), where NET formation markers (including circulating DNA-histone complexes and polymorphonuclear neutrophil elastase), can be important independent risk factors for retinopathy (74).

Glial cells, which are a part of the retinal neurovascular unit, are crucial to the inflammatory process of the retina. In order to maintain the health of the neuroretinal tissue, microglia monitor synaptic activity continuously and remove metabolic debris and dying cells through phagocytosis (75, 76). There are few branched microglia in the outer nuclear layer of the retina under physiological conditions. Under pathological conditions, such as in NPDR patients, a subretinal space and outer nuclear layer are invaded by microglia (77, 78). It has been demonstrated in a rodent DM model that 1 month after drug induction, microglia begin activating, 4 months later they invade the inner plexiform layer, and migrate to the outer nuclear and photoreceptor layers after 14-16 months (79). This suggests that activated microglia are highly proliferative and migratory, as they shift from a branched form to an amoeboid form (80–82). The early activation state of microglia is predominantly M2 anti-inflammatory, releasing anti-inflammatory cytokines, including interleukin-4 (IL-4), IL-10, IL-13, and TGF-β, which alleviate and enhance neuronal survival (83), serve to neutralize noxious stimuli and restore tissue homeostasis (84). However, prolonged abnormalities in the internal environment, or monocyte chemoattractant protein-1 (MCP-1) secreted by retinal neurons, cause an overreaction of microglia from M2 to M1 (85). This shift precedes neuronal cell death in the DR (86), releasing pro-inflammatory factors such as TNF-α, IL-1β, and ROS (87), among which TNF-α leads to apoptosis, increased retinal endothelial cells permeability, adhesion, and BRB permeability (88). Activated microglia also cause apoptotic imbalance in pericytes and induce the release of pro-inflammatory factors such as inducible nitric oxide synthase (iNOS) and TNFα from pericytes, which reduces cell viability and disrupts connexins in pericytes and endothelial cells, leading to severe leakage (89). This in turn exacerbates the pro-inflammatory effects of microglia CX3CL1 is a neuronal membrane-bound chemokine that can be hydrolyzed by proteases to soluble chemokines and activates CX3CR1 receptors on the surface of microglia. When CX3CR1 receptors are absent, microglia are activated and cause inflammation-mediated retinal neuronal damage through phagocytosis (90) and disrupt retinal vascular integrity (91) Fractalkine (FKN)/CX3CR1 regulates microglia activation in central nervous system diseases. FKN inhibits the NF-B pathway and activates the Nrf2 pathway in retinal microglia, thereby reducing inflammation-associated cytokines and ROS production and protecting the retina from diabetic damage (92). Kdm6a is a histone demethylase that removes the trimethyl group of histone H3K27. If Kdm6a expression is increased in diabetic mouse retinal microglia/macrophages, it can be exacerbated by promoting Lcn2 expression and impairing glycolytic production in photoreceptors (93).

By limiting the diffusion of excitatory neurotransmitters (*e.g.*, glutamate) and preventing excitotoxicity, MGCs control angiogenesis and regulate retinal blood flow. In addition to redistributing ions, regulating nutrient supply, and recycling retinal vitamin A, spatial buffering is also utilized by MGCs (94). In the diabetic state, there is increased expression of glial cell pro-fibrillary acidic protein, a marker of MGC activation (95), which promotes the release of many growth factors and inflammatory cytokines (96), such as VEGF and IL-1β (97). Some of the inflammatory cytokines released by activated MGCs can act back on MGCs to stimulate their production of more inflammatory suggesting that activated MGCs have the effect of amplifying the inflammatory response to DR (98). The mechanism of action as far as relevant can be considered is that the VEGF signal released from MGCs may act as a neuroprotective mechanism in the early stages of DR (99). As the inflammatory response persists, the VEGF released from activated MGCs acts as a pro-inflammatory cytokine, stimulating the production of more inflammatory cytokines and exacerbating the inflammatory response, which is the main cause of the inflammatory response and vasculopathy in DR (100). DM-related retinal inflammation is induced by CD40 by releasing ATP from MGCs, which activates P2X7 purinergic receptors and upregulates the expression of inflammatory factors (101, 102). MGCs also promote retinal microglia migration and induce activated microglia infiltration by secreting CX3CL1 and upregulating CX3CR1 receptor expression (103).

### 3.3 OS plays a key role in the progression of early DR

OS occurs when the body’s oxidative and antioxidant functions are out of balance. Cell metabolism, proliferation, differentiation, and immune regulation require moderate to low levels of free radicals to function normally under normal conditions (104). In addition to non-mitochondrial sources induced by DM or hyperglycemia, hyperglycemia-induced ROS (105, 106) are believed to contribute significantly to the increase in OS. Oxygen radicals are physiologically generated by the mitochondrial electron transport chains and possess an extra electron that confers significant instability and high reactivity (107). Under conditions of hyperglycemia, glucose autoxidation or the cytoplasmic NADPH oxidase activity, as well as modulation of mitochondrial respiratory chain activity, leads to a significant increase in ROS production (108). Considering that the retina is the most oxygen-consuming tissue in the body, it is more vulnerable to damage by excess ROS. When ROS are produced in excess, retinal OS increases and mitochondrial function is disrupted, resulting in a number of impairments in retinal tissue cell function, including apoptosis of retinal capillary cells, which are primarily responsible for ROS generation in the retina and for superoxide production (109). OS disrupts communication between pericytes and endothelial cells, leading to the disruption of the BRB and other microangiopathies (21). The hyperglycemia-induced increase in ROS also leads to reduced glutamate uptake by MGCs. Transient receptor potential cation channel 6, a Ca^2+^-permeable cation channel sensitive to OS, is readily detected in MGCs and is highly expressed under HG conditions (110). Diabetic-related fibrotic diseases result from endothelial-mesenchymal transitions (EndMTs). A phenotypic shift towards EndMT is induced by the treatment of primary human retinal endothelial cells (HRECs) with HG (111). Matrix metalloproteinases (MMPs) are a broad class of zinc-dependent proteases that regulate major biological functions, including tissue repair and cell signaling (112). Among them, MMP-2 and MMP-9 are induced by excess ROS, which are significantly elevated in the retina as found in studies based on both DR patients and animal models (113–117). There is a vicious cycle of mitochondrial damage and MMP activation caused by mitochondrial OS, while MMPs negatively regulate mitochondrial function (118).

The mitochondria generate OS in DM because of their role as oxidative metabolism engines (119). As a result of oxidative phosphorylation, which is primarily carried out by mitochondria, the retina contains more than 75% of oxygen-using photoreceptors and more than 75% of ATP-producing mitochondria (120). DM results in a biphasic response to retinal mitochondria, characterized by early and transient activation, where oxidative damage to mitochondrial DNA can be compensated adequately (121, 122). Due to persistent hyperglycemia, mitochondrial respiration is reduced by NADH, glucose is over-degraded, electron transport chains are loaded more heavily, and mitochondria produce more oxidants, resulting in ROS overproduction (106, 123) Retinal cells’ normal physiological function is dependent on maintaining glucose homeostasis. Diabetic reticulum-mitochondrial communication is disrupted, resulting in dysregulated mitochondrial autophagy and impaired glycolysis in DR (124). Abnormal activation of GSK3β leads to the downregulation of active β-linked proteins leading to synaptic neurodegeneration in RGCs by inhibiting ROS scavenging enzymes, thereby triggering OS-driven mitochondrial damage ultimately causing diabetic retinal neurodegeneration (125). Increased expression of acid sphingomyelinase-dependent ceramide in diabetic rat retinal mitochondria, induced mitochondrial damage, as evidenced by increased loss of mitochondrial membrane potential (ΔΨm), increased mitochondrial mass, and increased fragmentation in mtDNA (126). In addition, fragmented mtDNA escapes from the mitochondria into the cytoplasm, where it binds to cyclic GMP-AMP synthase (cGAS) and IFN gene (STING) phosphorylation stimulators, then activates interferon regulatory factor 3 (IRF3) via an ERK1/2-Akt-tuberin-mTOR-dependent pathway (127).

### 3.4 Abnormalities in lipid metabolism affect the onset and progression of early DR

A cohort study found that approximately 6.0% of DM patients taking lipid-lowering medications were diagnosed with DR at diagnosis, compared to 6.5% of DM patients not taking lipid-lowering medications. Lipids are one of the major risk factors for DR development and progression (128). Fenofibrate, an orally active PPAR-alpha agonist, was found to reduce the prevalence of DR by 31%-40% in the Fenofibrate Intervention and Event Lowering in Diabetes and the Action to Control Cardiovascular Risk in Diabetes, ACCORD studies (129). In contrast, cholesterol and common lipids showed no significant correlation with the development of DR, according to the Wisconsin Epidemiological Study of DR (130). It is pertinent to note that apolipoproteins or serum lipoprotein complexes, play a significant role in lipids and amphiphilic molecules. In addition to regulating lipoprotein transport and distribution, apolipoproteins facilitate lipoprotein binding to cell surface receptors. They aid in solubilizing hydrophobic lipids, improve lipid uptake by cells, and act as enzymatic cofactors (131). 3,4-hydroxybutyric acid can be an independent risk marker for progression to DR when its levels are more closely related to the DR (132). There are no novel findings from lipid testing in NPDR, especially in patients with T2DM. The correlation between DR and serum Lp(a) levels is evident and is independently correlated with serum Lp(a) levels in T2DM patients (133). Some of these biomarkers can be used to predict the development and severity of DR, such as apoE, apoC-II and apoC-III, and apoE/apoC-II, while apoA-I and apoA-II contribute to the prevention of prevent DR (134, 135). This also suggests that our subsequent studies can delve into the pathogenesis of DR around apolipoproteins.

### 3.5 circRNA/miRNA/lncRNA play an important role in early DR

The role of various RNAs in the cytoplasm is important in the onset and development of DR, especially circular RNA (circRNA) (136), microRNAs (miRNAs) (137), and long-stranded non-coding RNA (lncRNA) (138).

First, for circRNA, molecules in a closed-loop structure, circRNA are more stable than their linear transcripts (139), acting as miRNA sponges in cells, which can derepress miRNAs from their target genes and elevate the expression level of target genes (140). CircaRNA-ZNF532 functions as a miR-29a-3p sponge, regulating NG2, LOXL2, and CDK2 expression in the diabetic state, reducing chronic retinal inflammation and peripapillary degeneration (141, 142). When stimulated by a HG state, CircaRNA-ZNF532 promotes the expression of NG2, LOXL2, and CDK2, reducing chronic retinal inflammation as well as the degradation of the peripheral retinal pigment epithelium. The silencing of cZNF609 was proven to attenuate capillary degeneration, inflammation, and retinal vascular leakage in diabetic rats (143). The retina of diabetic mouse and diabetic subjects express cPWWP2A in response to stress induction. The silencing of cPWWP2A could increase retinal vascular leakage, pericyte loss, and acellular vessels and elevated levels of IL-2 interleukin-6 (IL-6), TNF-α, VEGF and MCP-1 (144). What’s more, CirHIPK3, found in the cytoplasm of HRVEC, downregulates miR-30a-3p activity, which upregulates VEGFC, FZD4, and WNT2 expression, resulting in abnormal proliferation, migration, and tubular formation (145). In ARPE-19 cells, HG increased the expression of hsa_circ_0041795 and decreased the expression of miR-646. Conversly by silencing hsa_circ_0041795, ARPE-199 cells showed enhanced proliferation and reduced apoptosis, were associated with reduced inflammatory responses due to the inflammatory factors TNF-α, IL-1β and IL-6 (146).

For miRNAs, a class of non-coding single-stranded RNA molecules of approximately 22 nucleotides in length encoded by endogenous genes, are involved in the regulation of almost all cellular activities, including cell proliferation, differentiation, and apoptosis (147). It has been shown that miRNAs can be used as potential diagnostic markers for DR (148, 149), including miRNAs (has-let-7a-5p, has-miR-novel-chr5_15976 and has-miR-28-3p), among others (150). These miRNAs can be used as biomarkers for DR stratification, encompassing all stages from early NPDR to late PDR (has-miR-195, has-miR-20a-5, has-miR-20b-5, and has-miR-451a) (151). MiR-195 can target Smurf2, increase the expression of YY1, VEGFA, and Snail1, and promote HG-induced EMT and cell permeability (152). Based on these evidences, hyperglycemia induces the downregulation of miR-15a and miR-16 and induces pro-inflammatory signaling pathways of IL-1β, TNF-α, and NF-κB (153). miR-30a activates retinal microglia in an NLRP3-dependent manner and promotes DR progression (154). Stimulation of HREC by HG increases miR-34a expression and accelerates cellular senescence and mitochondrial dysfunction (153). miR-146a can be activated in trans by the NF-κB pathway, and increased levels of miR-146a expression also hurt IL-1R/TLR-mediated NF-κB activation, which also promotes inflammation within the retina (155, 156). Low miR-200a was detected in both *in vivo* and *in vitro* DR models. Thus, miR-200a can be used as a potential therapeutic target by downregulating LIM structural domain protein 1 (PDLIM1) in DR to increase cell viability, alleviate the apoptotic state and significantly reduce cell migration in HG-treated HRMEC (157). Besides, miR-20b-5p was upregulated in diabetic rats and human retinal microvascular endothelial cells (HRMECs). The miR-20b-5p inhibitors increased the expression of tight junction-related proteins, such as occluding small band 1 (ZO-1), occluding, and claudin-5, which may lead to increased BRB permeability, microvascular leakage, and retinal damage (158). Under the HG state, miR-365 downregulates TIMP3 expression to promote retinal OS and affect gliosis in MGCs and exacerbate DR disease (159). In HG-stimulated ARPE-19 cells, the expression of miR-455-5p was significantly downregulated. Furthermore, increased expression of miR-455-5p enhanced cell viability and inhibited HG-induced apoptosis and OS injury, manifesting as reduced intracellular ROS and malondialdehyde production and NADPH oxidase 4 expression, as well as suppressed inflammatory response, including inhibition of IL-1β, IL-6 and tumor necrosis factor-α secretion (160). miR-486-3P inhibits the TLR4/NF-κB axis, protects MGCs from OS, inflammation, and apoptosis in the HG state (161). In the high-glucose hyperglycemic state, miR-495g promotes ganglion cell apoptosis, possibly by regulating Notch1 to interfere with PTEN/Akt signaling transmission (162).

In addition, for lncRNAs, most of which are catalyzed by RNA polymerase II for transcription, regulate gene expression mainly at the pre-transcriptional and post-transcriptional levels (163, 164). In ARPE-19 cells treated with HG, the expression of lncRNA small nucleolar RNA host gene 1 (SNHG1) was significantly increased. While silencing SNHG1 reduced the expression of inflammatory factors such as IL-6 and IL-1β, inhibited migration and proliferation, increased the expression of E-cadherin and ZO-1, and promoted apoptotic cells in ARPE-19 (165). lncRNA SNHG7 inhibited HG-induced endothelial mesenchymal transition (EndMT), and miR-34a-5p overexpression reverses this effect (166). SNHG16 is also upregulated in HG-induced vascular endothelial cells and activates the NF-kB pathway via miR-146a-5p/IRAK1 and miR-7-5p/IRS1, which is triggered by the PI3K/AKT pathway and promotes hrMEC dysfunction (166). To prevent HG-induced inflammatory factors produced by HMREC, caspase-3/7, and apoptosis, miR-19b is negatively regulated by MEG3 (167). lncRNA (VEAL2) is capable of regulating endothelial cell permeability, primarily by modulating the effects of PRKCB2-mediated endothelial ligand protein conversion, thereby reducing DR’s hyperpermeability in the hyperglycaemic HUVEC model (168). lncRNA H19 is downregulated in high-glucose conditions and can inhibit EndMT via TGF-β independent of Smad (169). In diabetic retina and HG-stimulated REC, increased expression of lncRNA HOTAIR binds LSD1, represses VE-calmodulin transcription by reducing the level of H3K4me3 on its promoter, and facilitates transcription factor HIF1α-mediated VEGFA transcriptional activation, leading to REC dysfunction (170). LncRNA X inactive specific transcript (XIST) directly binds to and inhibits has-miR-21-5p expression in HG-injured ARPE-19 cells, while hsa-miR-21-5p upregulation reverses the protective effect of XIST in HG-injured ARPE-19 cells (171).

### 3.6 Involvement of microorganism in early DR pathological changes

Since the establishment of the Human Microbiome Project in 2007, researchers have accumulated a comprehensive understanding of microbes and their function in maintaining homeostatic events, including their contribution to disease progression. It has been shown in rodent and human studies that gut biogenesis plays a role in susceptibility and development of obesity and T2DM (172, 173). According to subsequent studies, gut microbiota, especially short-chain fatty acids and small molecule components like secondary bile acids and triethylamines, are associated with chronic inflammation and immune system imbalances (174). However, for diabetic complications, the relationship is less clear (175). In a db/db mouse model of diabetes, intermittent fasting (IF) reduced the development of retinopathy, and these benefits were associated with an increase in Firmicutes but a decrease in Bacteroidetes (176). Researchers are increasingly coming to this conclusion that there is a link between gut microbiota and retinal disease, and that the microbiota-gut-retinal axis may play a role.

Firstly, it was shown that, unlike healthy controls (HC), the microbial richness of the intestinal flora in DR was higher than normal, mainly in the form of increased levels of Bacillus spp, Macrobacterium spp, Rachnoclostridium spp, and Alistipes, and reduced levels of Blautia, Eubacterium_hallii, Dorea Collinsella, and Romboutsia (177). Analysis of specimens from diabetic populations with and without DR by metagenomics, 293460 unique genes were identified in the non-DR group, compared to 283,235 unique genes in the DR group, and with regard to phylum levels, the DR group had a decrease in Actinobacteria but an increase in Bacteroides. In terms of genus-level reduction, bifidobacteria, and lactobacilli were reduced (178). Additionally, two clinical studies examined flora distribution in normal populations, diabetic patients without DR, and diabetic patients with DR. In one study, 16S rDNA analysis revealed a reduction in Akkermansia abundance in patients with DR and an increase in Lachnospira and Romboutsia abundance in healthy individuals. DR patients were enriched with Prevotella, while DM patients were enriched with Bacillus, Veillonella, and Pantoea (179). Another study showed reduced alpha and beta diversity in both DM and DR groups compared to the healthy group, with increased levels of Bifidobacterium and Lactobacillus and reduced levels of E. coli, E. faecalis, Eubacterium_hallii_group, and Clostridium spp. in both DM and DR groups were observed. Compared to HC, Bacillariophyceae increased and DR decreased in DM patients (177).

In the decade since the Human Microbiome Project was first published, much attention and insight have been given to the role of the human microbiome in physiology and pathology. The eye is an organ that is constantly exposed to the external environment and attracts a variety of microorganisms, and a link has now been made between the microbiome and populations with ocular disease (180, 181). The alpha diversity of the ocular surface microbiome was previously reported to be more diverse in DM compared to the non-DM group. Studies have shown that pathogenic bacteria such as Enterobacteriaceae, Neisseriaceae, Escherichia-Shigella and Pseudomonas predominate in patients with DR. DR affects changes in the microbiome of the ocular surface. Furthermore, studies have shown that the microbiome composition in the gut and plasma may affect the interior of the eye and retina. Through the characteristic disruption of the BRB associated with DR, the consequent increase in retinal bacterial products may activate signaling pathways such as TLR and GPR81. This activation could further stimulate the local release of cytokines and VEGF, supporting a role for the gut-retinal axis in the pathogenesis of DR (182).

The causal relationship between DR and microorganisms, sequential and primary has not been clarified, so the follow-up still needs to carry out in-depth related research.

### 3.7 Cell death is central to the mechanism of early DR pathological changes

**Figure 3:**
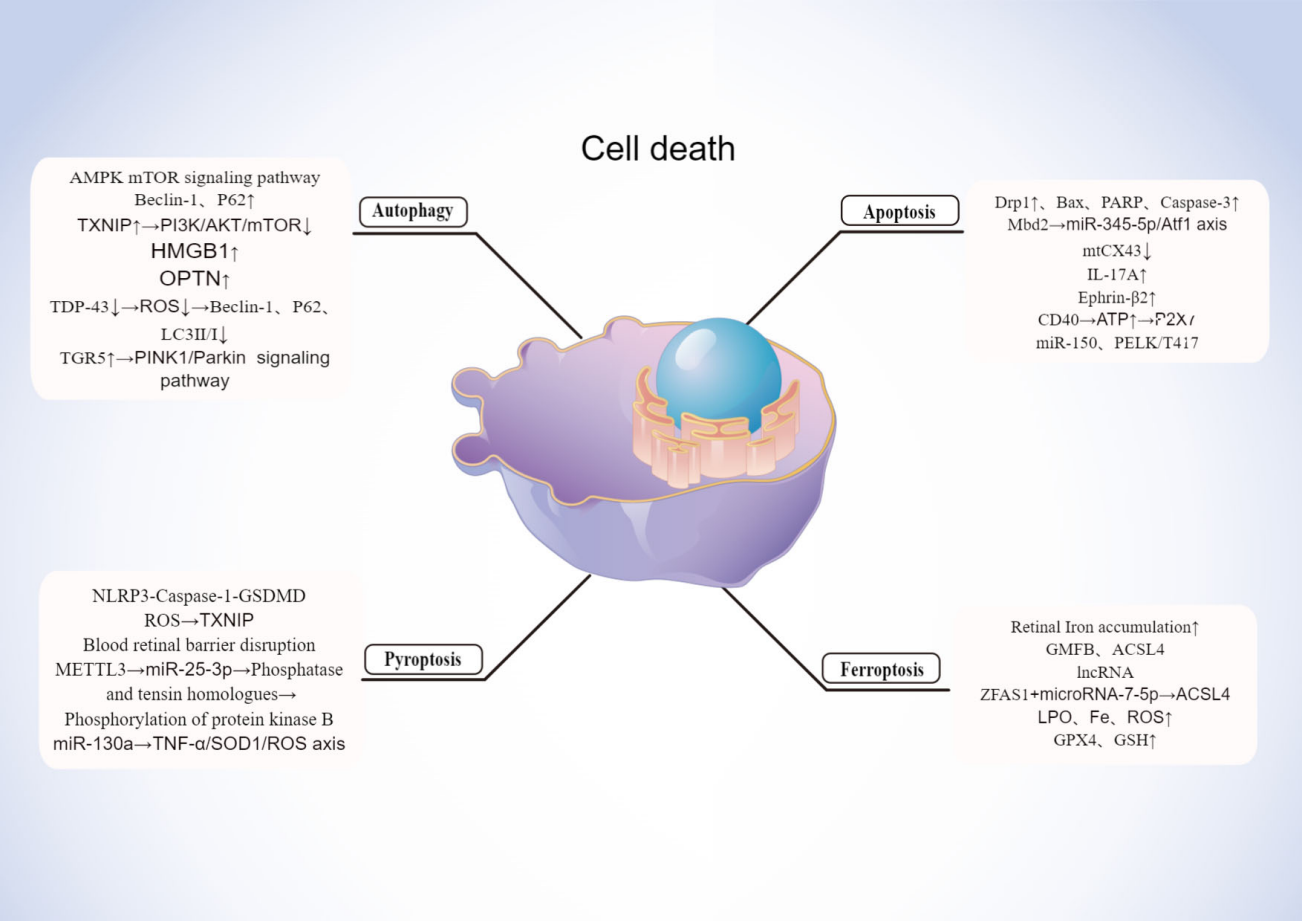
Mechanisms of cell death in DR. Created By Figdraw. HMGB, High mobility group box 1; ROS, Reactive oxygen species; OPTN, Optineurin locus.

With the advancement of research, multiple modes of cells death are now a hot topic of research and a future direction for the study of DR. Early pathological changes in DR include the death of pericytes, neurons, *etc.* Currently novel death modalities such as autophagy, ferroptosis, pyroptosis play a key role in the death of these cell, which is shown in **Figure 3**.

#### 3.7.1 Autophagy

Lysosomes degrade and recycle cellular components through autophagy, a catabolic process (183). Macroautophagy, microautophagy, and chaperone-mediated autophagy (CMA) are primarily classified by the pathway by which cellular material is transported to the lysosome (184). Autophagy is a pro-survival and anti-apoptotic adaptive response to moderate stress or in the early stages of DR (185). In contrast, under severe stress in the later stages of DR, dysfunctional autophagy leads to retinal cell death by apoptosis and exacerbates long-term damage, creating systemic overload (186, 187).

In addition to the lack of nutrients such as amino acids in particular, autophagy is closely connected to the AMPK and mTOR signaling pathways (188). Chronic hyperglycemia leads to mTOR inhibition, resulting in the dysregulation of autophagy (189). The autophagy-related proteins Beclin-1 and P62 are expressed more often in DR patients, and mTOR inhibition reduces autophagic damage to retinal cells (190). Under HG conditions, TXNIP knockdown reduced autophagy and apoptosis in rat MGCs. As a result of inhibiting the PI3K/AKT/mTOR signaling pathways, TXNIP positively regulates autophagy (191). The second factor that complicates the understanding of DR is autophagy, which promotes pericyte survival in early DR, while excessive autophagy damages the pericyte and results in necrosis. It has been shown that autophagy regulated by high mobility group box 1 (HMGB1) is involved in the development of DR. However, HMGB1 is involved in LMP via CTSB (histone B)-dependent pathway, but not CTSL (histone L)-dependent pathway. By knocking down HMGB1 expression, RPE cells were protected from apoptosis, autophagy was restored, inflammation factors were reduced, and VEGF expression was decreased in the early stage of DR (192). In vitreous fluid samples from DR and non-DR patients, 74 Loci out of 35 proteins also had different succinylation patterns, and the optineurin locus (OPTN K108 su) was significantly enriched by GO analysis based on biological process categories. In the rat model of DM induced with streptozotocin (STZ), primary rat RGCs, and R28 cells, OPTN was shown to undergo lysine succinylation in the retina of DR rats and OPTN K108 su mediated autophagic flux blockade at high concentrations, respectively (193). Zabel et al (90) discovered that HG increased ER stress with apoptosis, accumulated p62, and upregulated early and late-phase autophagy markers (LC3II/I, Beclin-1) in cultured rMC-1. Under HG circumstances, autophagy was inhibited, which enhanced the rate of rMC apoptosis (190). Third, dysfunction of the autophagic pathway leads to superoxide formation and apoptosis (194). ROS accumulation enhances activation of autophagy (195). The RNA-binding protein known as Transactivation Response DNA-Binding Protein of 43 (TDP-43) is a well-known member of the heterogeneous ribonucleoprotein family. TDP-43 is abundantly expressed in RGC-5 cells exposed to H_2_O_2_, and it has been shown to have a role in the etiology of neuronal degenerative disorders (196). TDP-43 inhibition greatly reduced the formation of intracellular ROS and H_2_O_2_-induced OS. Additionally, TDP-43 downregulation inhibited the expression of Beclin-1, p62, and LC3II/I as well as the H_2_O_2_-triggered autophagy. Histone deacetylase 6 (HDAC6) is no longer expressed as a result of TDP-43 suppression, and HDAC6 also lessens the inhibitory impact of TDP-43 downregulation on H_2_O_2_-induced autophagy and death. Therefore, by preventing HDAC6 in the DR, TDP-43 silencing might shield RGC-5 cells from OS-mediated autophagy and death (197). The stress-activated kinases JNK and p38 MAPK were more heavily phosphorylated in ARPE-19 cells grown in HG or hypoxia. Autophagy was suppressed by phosphorylating PERK and eIF2a, which both elevated the pro-apoptotic transcription factor CHOP (198). This experimental condition elevates ROS and disrupts tight junction integrity.

A particular type of autophagy called mitochondrial autophagy is in charge of regulating the amount and quality of mitochondria (199). Overexpression of PINK1 or Parkin can counteract HG’s inhibitory impact on cellular mitochondrial autophagy and proliferation as well as its encouragement of apoptosis (200). In addition, the effect of glucose on the retinal pigment epithelium (RPE) varies with dose (200). Autophagy protects cells from OS damage in the short term when they are exposed to HG, but in the long term, autophagy is inhibited and apoptosis is enhanced (201). HG-induced mitochondrial disruption plays a key role in promoting apoptosis in retinal vascular cells. Downregulation of the mitochondrial fission genes Fis1 and Drp1, which are overexpressed under HG conditions, prevents mitochondrial breakage, maintains mitochondrial function, and protects retinal endothelial cells from apoptosis, and reduces expression of the pro-apoptotic proteins Bax and cleaved cysteines 3 (202). In a study by Taki and colleagues in 2020, HG treatment (25 mmol/L, 48 h) was found to induce mitochondrial accumulation and upregulation of p62 in transformed mouse cone cell line, 661W cells. The PINK1-Parkin pathway and the BNIP3L-LC3 connection are both implicated in mitochondrial autophagy (201). By blocking Ca2+ -PKC/Drp1 signaling and promoting mitochondrial autophagy by upregulating the PINK1/Parkin signaling pathway, the bile acid G protein-coupled membrane receptor (TGR5), a new bile acid cell membrane receptor, improves vascular endothelial cell dysfunction in DR. Additionally, another research found that Drp1 promoted hexokinase (HK) 2 separation from mitochondria and HK2-PINK1/Parkin signaling, which in turn prevented mitochondrial autophagy (203).In retinal pigment epithelium (RPE), PINK1 knockdown reduces the amount of phosphorylated Parkin.

#### 3.7.2 Pyroptosis

Inflammation-induced pyroptotic cell death is characterized by the formation of pores in the cell membrane, rapid swelling, and rupture of the membrane, resulting in massive cytoplasm leakage (203, 204). Cell scorch death was defined in 2018 by the Nomenclature Committee on Cell Death as a process requiring large amounts of Gasdermin (GSDM) proteins to form pores in the plasma membrane. As a result of inflammatory cysteine-aspartic protease activation (cysteine-aspartic acid protease, caspase), regulated cell death (RCD) is often but not always triggered (205). A double-edged sword for the body is Pyroptosis: On one hand, it stimulates immune cells to eliminate infectious agents by releasing immunostimulatory cytokines (206). Conversely, scorch death as a form of cell death can impair tissue health and has a clear correlation with the pathophysiology and prognosis of inflammatory diseases, as evidenced by a growing number of studies.

It has now been established that there are two primary activation mechanisms for cell scorch death, one of which involves highly inflammatory lysis-induced cell death brought on by the cleavage of the GSDMD by activated caspase-1. Different pathogen-associated or damage-associated molecular patterns activate the relevant cytoplasmic inflammasome sensors, which are a number of NOD-like receptor (NLR) proteins, such as NLRP1, NLRP3, NLRC4, AIM2, and Pyrin, in the caspase-1-mediated classical inflammasome pathway. Activated caspase-1 then cleaves the protein GSDMD to form pores, releasing a significant amount of cellular contents and secreting more IL-1 and IL-18 extracellularly, leading to a severe inflammatory response. Caspase-1 and the junctional protein apoptosis-associated particulate protein (ASC) are then recruited to form inflammasomes. The second pathway, Caspase-4/5/11, is in charge of the non-classical cell scorch pathway and is triggered by the bacterial lipopolysaccharide, which directly activates either human or mouse caspase-4/5. Additionally, GSDMD is also broken down by activated caspase-11/4/5, which releases functional GSDMD-N terminal to create holes in the plasma membrane (207). Caspase-1 is triggered by NLRP3-ASC conjugates to cleave the pro-IL-1 and pro-IL-18 that are not yet active, unleashing the mature IL-1 and IL-18, and causing cellular scorch death (208). These data imply that GSDMD is both the ultimate executor of both activation pathways and a critical determinant of cell scorch (209).

Pericytes, endothelial cells, retinal neurons, microglia, and RPE cells (pathological alterations in the early stages of DR) have been demonstrated to be intimately associated to pyroptosis. Gan et al. (210) found that HG led to peripapillary cell loss in part due to NLRP3-Caspase-1-GSDMD-mediated cell scorch death. The HG environment also induced NLRP3 inflammatory vesicle-dependent cell scorch death in retinal microglia, leading to retinal neurovascular damage. It was also found that severe OS from excessive ROS accumulation was shown to be an important cause and exacerbation of DR, and HG induced an increase in reactive ROS in retinal microvascular endothelial cells, upregulating thioredoxin-interacting protein (TXNIP) expression and activating the classical scorch death pathway, leading to pyroptosis and increased retinal vascular permeability (211). A similar mechanism of pyroptosis is seen in the RPE. Damage to retinal microvascular endothelial cells and the RPE, which are the main cells that form the internal and external barriers of the blood retina, leads to disruption of blood retinal barrier function and exacerbates vascular leakage in DR (212). In addition, HG was found to inhibit RPE cell proliferation, promote apoptosis and pyroptosis in a time-dependent manner. Methyltransferase-like protein 3 (METTL3) controls N 6-methyladenosine modifications to control cell function and disease. METTL3 overexpression raises the levels of the miR-25-3p in RPE cells, which in turn negatively controls the expression of phosphatase and tensin homologs, encourages protein kinase B phosphorylation, and ultimately reduces the HG-induced RPE pyroptosis (213). In addition, upregulation of mi R-130a attenuated the toxic effects of HG on RPE cells by regulating TNF-α/SOD1/ROS axis-mediated RPE pyroptosis (214). All of the above findings confirm that the occurrence of pyroptosis is closely related to the pathological process in the early stages of DR patients.

#### 3.7.3 Ferroptosis

During ferroptosis, intracellular lipid ROS accumulate intracellularly, resulting in iron-dependent RCD. Disturbances in iron metabolism (*e.g.*, cysteine transport pathway, metabolic pathway, and lipid metabolism pathway) are the main cause of cellular ferroptosis (215, 216). It is not uncommon for mitochondrial wrinkleling, the reduction or loss of cristae, and membrane thickening to occur in a state of iron imbalance.

There is increasing evidence that ferroptosis is associated with the development of DR. In DM patients, iron levels in retinal tissue are increased due to factors such as hyperglycaemia and OS, which can lead to iron deposition and cellular damage. In addition, lncRNA can regulate the process of iron death affecting the development of DR. Iron atrophy was suppressed if zinc finger antisense protein 1 (ZFAS1) was inhibited, and in HG cultured hRECs, ZFAS1 was upregulated leading to ferroptosis. MicroRNA-7-5p (miR-7-5p) is a competitive endogenous RNA that can act as a competitive endogenous RNA by competing with it and regulating the expression of its downstream molecule, acyl-coenzyme A synthase long-chain family member 4, which is now identified as a classical driver of ferroptosis (217). In addition, some drugs that inhibit or promote ferroptosis can also affect the progression of DR.

Even in the early stages of DR, ferroptosis is present. According to one study, DR patients’ GPX4 and GSH concentrations were considerably reduced, but their LPO, Fe, and ROS concentrations were noticeably greater. The NPDR group showed greater LPO, Fe, and ROS concentrations compared to the PDR group, while having lower GPX4 and GSH concentrations. Ferroptosis-related biomarkers had cumulative accuracy in NPDR, according to the ROC curve (218). Additionally, individuals with early DR showed elevated levels of the neurodegenerative factor glial maturation factor (GMFB) in their vitreous. Large quantities of GMFB protein may be produced in the vitreous in an HG environment, which may cause the ATP enzyme ATP6V1A to be diverted from the lysosome, block its assembly, and alkalize the lysosome in RPE cells (219). The lysosome eventually breaks down the ACSL4 protein once it is identified by the chaperone-mediated autophagy receptor HSC70. It builds up as a result of abnormalities in the autophagy-lysosome degradation pathway, which triggers the creation of deadly lipid species and eventually causes ferroptosis in RPE cells (220).

#### 3.7.4 Apoptosis

During apoptosis, cells cease to grow and divide as they undergo a process that results in their controlled death in the environment with their contents spilling out. Apoptosis is a form of programmed cell death responsible for maintaining the homeostasis of the internal environment, which is induced in retinal cells by the HG environment of DR. Retinal endothelial cells are affected by mitochondrial dysfunction and apoptosis caused by HG-induced overexpression of Drp1. The same was demonstrated in animal studies as well. Diabetic Drp1 +/- mice had increased levels of Bax, cleaved PARP, and cleaved caspase-3 expression compared to diabetic Drp1 +/- mice (221). Methyl-CpG binding domain protein 2 (Mbd2) mediates HG-induced apoptosis in RGC through the regulation of miRNAs, and HG-induced overexpression of Mbd2 in the retina is partially responsible for apoptosis in retinal neuronal cells and acts through the miR-345-5p/Atf1 axis (222). HG-induced mitochondrial connexin 43 (mtCx43) levels are reduced to promote DR-associated mitochondrial fragmentation of retinal endothelial cells causing retinal endothelial apoptosis (223). In addition, the pro- IL-17A exacerbates HG-induced retinal Müller cells activation and dysfunction *in vitro* and serves to promote retinal neuronal death during the DR process (224).

DM significantly increases Ephrin-B2 expression in diabetic retina and HRP, increasing Ephrin-B2 signaling in the pericytes, and leading to inflammation and apoptosis in retinal vessels (225). DR is not only a vascular disease, but also a more complex neurodegenerative disease. Axonal degeneration, glial abnormalities, and neuronal cell death are pathogenic mechanisms of diabetic retinal neuronal abnormalities. When retinal Müller glial cells express CD40, the secreted ATP activates P2X7 receptors, leading to the release of pro-inflammatory cytokines by monocytes/macrophages/microglia, and to the death of retinal endothelial cells. Capillary degeneration and retinal ischemia result from endothelial cell death caused by the CD40-ATP-P2X7 pathway (226). P2X7R plays an important role to regulate BRB integrity. Photoreceptor cells die shortly after DM begins, resulting in retinal dysfunction and microvascular complications that can cause vision loss. PELK1T417 is phosphorylated by miR-150 in the T2DM retina, thereby targeting ETS structural domain transcription factor (ELK1). A key step in diabetic-induced photoreceptor cell apoptosis is the translocation of pELK1 T417 into the nucleus (227).

## 4 Therapeutic strategies in the early stages of DR and the exploration of emerging pharmacological agents

**Figure 4:**
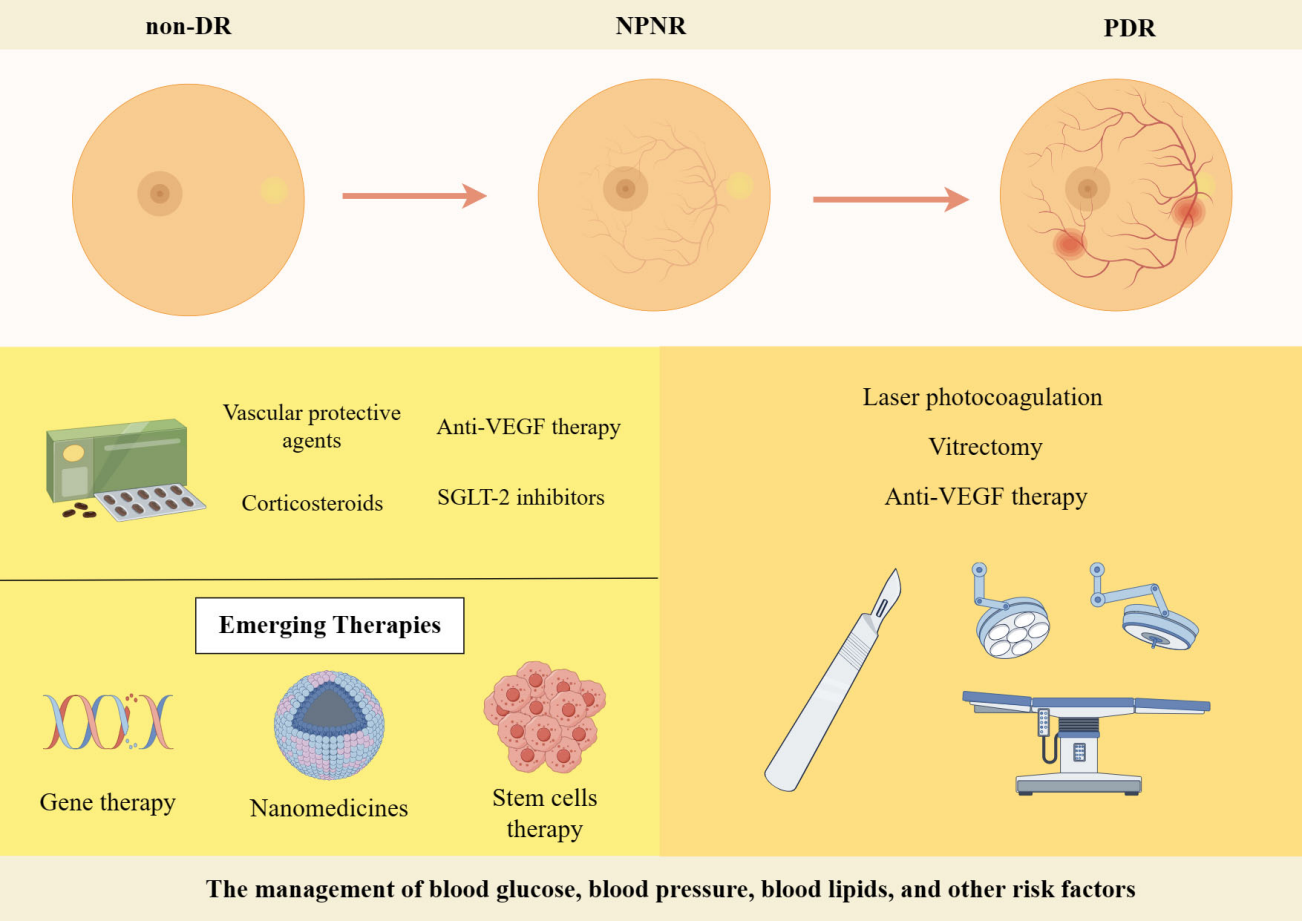
Treatment strategies for DR. Created By Figdraw. SGLT-2, Sodium-Glucose Linked Transporterinhibitors-2; VEGF, vascular endothelial growth factor.

The management of blood glucose, blood pressure, blood lipids, and other risk factors is crucial in the treatment of different stages of DR. Regular fundus screening should be conducted throughout the process. The treatment of PDR primarily relies on laser photocoagulation, vitrectomy, or anti-VEGF therapy when comparing early-stage DR treatments with full-onset PDR treatments (228). The management of NPDR primarily relies on pharmacological interventions (**Figure 4**). In recent years, with the increasing focus on research into DR, numerous novel therapies and drugs have been developed that exhibit promising results in both preclinical and clinical trials. This article highlights current therapeutic strategies as well as the exploration of emerging drugs for early-stage DR, which is shown in **Table 2**.

**Table 2:** Therapeutic strategies in the early stages of DR and emerging pharmacological agents.

Treatment	Drug	Study design	Function	Reference
SGLT-2 inhibitors	Dapagliflozin	RCT	A short-term treatment with dapagliflozin may central retinal thickness.	(229)
Vascular protective agents	Calcium Dobesilate	RCT	Calcium Dobesilate can ameliorate retinal microaneurysms, haemorrhage, and exudation in the fundus, as well as reducing whole blood viscosity, plasma viscosity, and blood cholesterol levels.	(230, 231)
Anti-VEGF therapy	Abciximab	RCT	Aflibercept treatment resulted in a significant reduction of eyes with vision-threatening complications and central involvement of DME.	(232)
Corticosteroids	Dexamethasone	RCT/meta	Significant improvements in visual acuity and central macular thickness could be achieved through dexamethasone implantation.	(233, 234)
Gene therapy	ADVM-022	A phase II clinical trial	A single intravitreal administration of ADVM-022 may provide a safe and effective long-term treatment option for neovascular age-related macular degeneration and DME, and may ultimately improve patients’ visual outcomes.	(235)
Stem cells therapy	Autologous Bone Marrow Mesenchymal Stem Cell Transplantation	Non-randomized, open-label, prospective, no-placebo, single-center study	Patients with severe NPDR and mild PDR showed a tendency towards improvement in visual acuity, visual electrophysiological indices, macular thickness, blood glucose levels, inflammatory response, as well as a better safety profile after receiving autologous bone marrow MSC treatment.	(236)
Nanomedicines	Dexamethasone gamma-cyclodextrin nanoparticle eye drops	RCT	Topical dexamethasone gamma-cyclodextrin nanoparticle eye drops have the potential to significantly enhance visual acuity and reduce macular thickness in patients.	(237)

Firstly, significant progress has been made in the development of various drugs targeting blood glucose, blood pressure and lipids to control risk factors for DR and improve metabolic disorders. Some novel drugs, such as Sodium-Glucose Linked Transporterinhibitors-2 (SGLT-2), Glucagon-like peptide 1 receptor agonists and fenofibrate have demonstrated efficacy in controlling blood glucose levels, lowering lipid levels and improving DR (229). DR progresses from NPDR to PDR, which is characterized by the growth of abnormal blood vessels in the retina. Calcium Dobesilate has demonstrated efficacy in protecting blood vessels and improving local circulation, rendering it a promising therapeutic option for DR. A randomized, double-blind, controlled clinical trial revealed that administering 2 g of calcium dobesilate daily over the course of two years was significantly more effective than placebo at preventing blood-retinal barrier disruption in patients with T2DM who had early-stage DR (238). Zhang et al. (230) demonstrated the efficacy of calcium dobesilate in ameliorating retinal microaneurysms, haemorrhage, and exudation in the fundus, as well as reducing whole blood viscosity, plasma viscosity, and blood cholesterol levels (231). Nevertheless, further validation through a large-scale, multicentre, randomized double-blind controlled study is still warranted. The primary etiology of visual impairment in diabetic patients is attributed to the development of DME, which arises from vascular permeability and disruption of the BRB (239). Anti-VEGF therapy has been shown to significantly improve the severity of non-proliferative DR in patients, as evidenced by recent prospective clinical trials (232, 240). The FDA has approved intravitreal abciximab for the treatment of non-proliferative DR, PDR and DME (241). However, it has been demonstrated that intravitreal injection of bevacizumab leads to a reduction in macular thickness in patients without DR or NPDR and without macular edema during the early postoperative period. Nevertheless, this effect is not sustained at 3 months, and no significant differences from controls are observed throughout the follow-up period (242). Intravitreal corticosteroid injections are a viable treatment option for persistent DME or cases where first-line treatments such as anti-vascular endothelial growth factor injections are not effective. The current corticosteroids utilized for the treatment of retinopathy comprise tretinoin, dexamethasone and fluocinonide. In a 16-wk randomized controlled trial involving 140 patients with DME, intravitreal implantation of dexamethasone demonstrated significant improvement in visual acuity and central macular thickness compared to the group receiving intravitreal injection of ranibizumab (233). Yuan et al. (234), conversely, conducted a Meta-analysis of the findings from 10 clinical trials investigating the efficacy of dexamethasone implantation in patients with refractory DME. Their analysis revealed that significant improvements in visual acuity and central macular thickness could be achieved through dexamethasone implantation. However, Yilmaz et al. (243) discovered that while trimethoprim injections for DR resulted in greater visual acuity improvement at 3 months, there was no significant effect observed at the 6-months mark. Additionally, patients who received trimethoprim injections experienced significantly higher IOP levels both at the 3 and 6-months follow-up periods.

In recent years, the advancement of gene technology has brought gene therapy for DR into the forefront of research (244). Currently, there are two types of gene therapy being studied for DR: Those that inhibit retinal neovascularization and those that aim to protect the retinal vascular nerve unit from damage. Gene therapy necessitates the utilization of a vector, predominantly adeno-associated virus (AAV) vectors in clinical trials. RGX-314 is administered via an AAV8 vector that encodes a monoclonal antibody fragment (anti-VEGF fab). This fragment effectively neutralizes VEGF activity and obstructs the VEGF pathway, functioning similarly to ranibizumab. In the INFINITY study (NCT: 04418427), a phase II clinical trial of ADVM-022, it was observed that ADVM-022 exhibited favorable therapeutic efficacy in patients with DME as compared to the control drug abciximab. At 12 weeks of treatment, nearly half of the patients demonstrated an improvement in DR severity scores by ≥ 2 steps. However, it is noteworthy that a higher incidence of intraocular inflammation was reported among subjects post-treatment. Apoptosis of retinal neuronal cells, capillary endothelial cells, and pericytes under conditions of hyperglycemic hypoxia, as well as progressive loss of retinal neurovascular unit integrity, give rise to a cascade of pathological changes in the retina. Stem cells serve as the cellular source within an organism and possess remarkable potential for multidirectional differentiation, homing, regeneration, and secretion of growth factors. These attributes hold significant clinical value in the treatment of DR (245). A study conducted by Gu et al. (236) assessed the safety and efficacy of intravenous infusion of autologous bone marrow MSCs in treating DR. The results indicated that patients with severe NPDR and mild PDR showed a tendency towards improvement in visual acuity, visual electrophysiological indices, macular thickness, blood glucose levels, inflammatory response, as well as a better safety profile after receiving autologous bone marrow MSC treatment. Although stem cell therapies hold great potential for both early and late-stage DR treatment, high-quality randomized controlled trials are still necessary to identify the most suitable DR patients for these therapies before stem cell transplantation can become a routine treatment option. Nanomedicines have emerged as a prominent research area due to their exceptional targeting properties, low toxicity, and superior pharmacokinetic characteristics (246), making them widely used in the treatment of various diseases. A prospective randomized controlled trial conducted by Ohira et al. (237) demonstrated that topical dexamethasone gamma-cyclodextrin nanoparticle eye drops have the potential to significantly enhance visual acuity and reduce macular thickness in patients with DME, exhibiting effects comparable to those of tretinoin. Although nanomedicines offer unique advantages, further comprehensive studies are required for their application in the clinical treatment of DR.

However, despite the demonstrated efficacy of these drugs in preclinical and clinical trials, several challenges remain. For instance, the long-term safety and effects of many drugs have yet to be established, while small sample sizes and short trial durations characterize many studies. Furthermore, given the complex pathophysiological mechanisms underlying DR, it is possible that single-target drugs may not achieve optimal therapeutic outcomes. Future research therefore needs to build on the understanding of the pathophysiological mechanisms of DR to develop more effective and safer drugs to address the therapeutic needs of this serious complication.

## 5 Conclusion

DR is one of the most common complications in people with DM. Microvascular diseases are traditionally considered to be the cause of this condition. The American Diabetes Association describes it as neurovascular complication with a high degree of tissue specificity (247). Prior to microvascular damage, the glial cell unit experiences early changes. A significant portion of DR is caused by disruption of both the BRB and retinal neurovascular unit. This review summarizes the results of recent basic research around early pathological alterations in DR. We include mechanisms relating to leukocyte adhesion, neutrophil extracellular traps, microglial activation, pyroptosis and ferroptosis, as well as previous classical signaling pathways and cutting-edge directions for future development.Of course, there are also emerging modes of cell death, such as efferocytosis and cuproptosis, which have not been investigated in the context of diabetic retinopathy and may serve as potential areas for future research on DR.

Although the early stages are critical for intervention in DR, there are currently no targeted therapeutic drugs for this stage. The development of effective treatments for DR requires a clear understanding of the pathological and molecular mechanisms responsible for early DR abnormalities. We have discussed the pathological alterations and molecular mechanisms of early-stage DR, which may provide potential treatment targets as well as promising directions for future research. In spite of the useful information has been obtained from cellular and animal experiments, much work remains to be done to develop interventions for early-stage DR in clinical studies.

## Author contributions

WS: Writing – review & editing. XA: Writing – review & editing. YZ: Writing – review & editing. XZ: Writing – review & editing. YS: Writing – review & editing. CY: Writing – review & editing. XK: Writing – review & editing. LJ: Writing – review & editing. HJ: Writing – original draft. FL: Writing – original draft.
